# Correction: Regulating toxic chemicals for public and environmental health

**DOI:** 10.1371/journal.pbio.1002619

**Published:** 2018-01-25

**Authors:** The PLOS Biology Staff

## Notice of Republication

This article was republished on January 10, 2018, to correct errors in the references. The publisher apologizes for the errors. Please download this article again to view the correct version. The originally published, uncorrected article and the republished, corrected articles are provided here for reference.

## Supporting information

S1 File Originally published, uncorrected article. (PDF)

S2 File Republished, corrected article. (PDF)
