# Supplementary material for: Correction: Regulating toxic chemicals for public and environmental health
Source: PLoS Biol. 2018 Jan 25;16(1):e1002619. doi: 10.1371/journal.pbio.1002619 (PMC5784888; doi:10.1371/journal.pbio.1002619)
Supplement: S2 File — (PDF) [file pbio.1002619.s002.pdf]

EDITORIAL

Regulating toxic chemicals for public and environmental health

Liza Gross[1]*, **Linda S. Birnbaum**[2]*

1 Public Library of Science, San Francisco, California, United States of America, **2** National Institutes of Health, Department of Health and Human Services, Research Triangle Park, North Carolina, United States of America

* lgross@plos.org (LG); birnbaumls@niehs.nih.gov (LSB)

This Editorial is part of the *Challenges in Environmental Health: Closing the Gap between Evidence and Regulations Collection.*

By the time President Gerald Ford signed the United States Toxic Substances Control Act in the fall of 1976, tens of thousands of synthetic chemicals had entered world markets with no evidence of their safety. Ford's signing statement described a law giving the Environmental Protection Agency (EPA) broad regulatory authority to require toxicity testing and reporting to determine whether the chemicals posed risks. "If a chemical is found to present a danger to health or the environment," Ford promised, "appropriate regulatory action can be taken before it is too late to undo the damage."

That's not what happened. The 60,000-plus chemicals already in commerce were grandfathered into the law on the assumption that they were safe. And the EPA faced numerous hurdles, including pushback from the chemical industry, that undermined its ability to implement the law. Congress finally revised the law last year, with the Frank R. Lautenberg Chemical Safety for the 21st Century Act, to bolster the EPA's regulatory authority. Over the decades that US policy on chemicals stagnated, scientists documented the damage whole classes of chemicals inflicted on living organisms and the environment that sustains them. Although we still have safety data on just a fraction of the 85,000-plus chemicals now approved for use in commerce, we know from field, wildlife, and epidemiology studies that exposures to environmental chemicals are ubiquitous. Hazardous chemicals enter the environment from the factories where they're made and added to a dizzying array of consumer products—including mattresses, computers, cookware, and plastic baby cups to name a few—and from landfills overflowing with our cast-offs. They drift into homes from nearby agricultural fields and taint our drinking water and food. Today, hundreds of industrial chemicals contaminate the blood and urine of nearly every person tested, in the US and beyond.

In the decades since Ford promised a robust policy to regulate potentially hazardous chemicals, evidence has emerged that chemicals in widespread use can cause cancer and other chronic diseases, damage reproductive systems, and harm developing brains at low levels of exposure once believed to be harmless. Such exposures pose unique risks to children at critical windows of development—risks that existing regulations fail to consider. To address these issues, *PLOS Biology* is publishing a special collection of seven articles, Challenges in Environmental Health: Closing the Gap between Evidence and Regulations, that focus on US chemical policy [1].

In commissioning the collection, we aimed to reveal barriers to developing health-protective policies not only when the scientific evidence of harm is clear but also when it is uncertain. We sought to explore the technical challenges involved in determining how the hundreds of

OPEN ACCESS

Citation: Gross L, Birnbaum LS (2017) Regulating toxic chemicals for public and environmental health. PLoS Biol 15(12): e2004814. https://doi.org/10.1371/journal.pbio.2004814

Published: December 18, 2017

Copyright: This is an open access article, free of all copyright, and may be freely reproduced, distributed, transmitted, modified, built upon, or otherwise used by anyone for any lawful purpose. The work is made available under the Creative Commons CC0 public domain dedication.

Funding: The authors received no specific funding for this work.

Competing interests: I have read the journal's policy and the authors of this manuscript have the following competing interests. Liza Gross is a current paid employee at the Public Library of Science.

Provenance: Not commissioned; not externally peer reviewed.

chemicals we carry in our bodies affect health. These challenges include ascertaining exposures and impacts of short-lived compounds; identifying chemicals that pose unique risks to the developing fetus; and assessing the risk of chemicals that cause proportionately more harm at the lowest levels of exposure in violation of longstanding toxicology principles. We asked authors to consider these issues within their field of expertise and to suggest ways to bridge the gap between evidence and policy.

Several articles explore the failure of regulations to keep hazardous chemicals from polluting our food, air, and drinking water. Maricel Maffini and her colleagues describe the failure of regulators to account for health risks associated with the thousands of chemicals introduced into the food system since 1958, when Congress authorized the Food and Drug Administration to ensure the safety of substances added to food [2]. Sheldon Krimsky argues that an "unreasonable risk" standard to assess industrial chemicals in both the original and revised Toxic Substances Control Acts has imposed enormous data gathering and resource demands on the EPA, and ultimately hobbled the agency's ability to regulate [3].

But as Bruce Lanphear points out, no policy will protect public health if it doesn't account for the upending of one of toxicology's most fundamental precepts: the dose makes the poison [4]. Over the past three decades, Lanphear notes, evidence from some of the most extensively studied toxic chemicals—including lead, asbestos, tobacco, and benzene—shows that some chemicals are most toxic at the lowest levels of exposure. Yet regulations still assume that toxic effects emerge at a threshold level and increase with the dose. Protecting public health, Lanphear argues, requires rethinking basic assumptions about how agencies regulate chemicals.

Existing policy also fails to account for the fact that individuals are exposed to multiple chemicals every day, from the point of conception to the end of life. As Joseph Braun and Kimberly Gray note, epidemiologists are working to determine the full range of chemicals we carry in our bodies and how they affect health [5]. Toward that end, they're developing new methods to accurately estimate exposure to chemical mixtures, identify periods of heightened vulnerability, and flag chemicals that are particularly hazardous to children's health.

But having solid scientific evidence that a chemical causes harm, even to our children, is no guarantee that policymakers will act accordingly, Leo Trasande argues [6]. Using the failure to ban the pesticide chlorpyrifos as a case study, Trasande lays out the evidence that organophosphate pesticides like chlorpyrifos can damage the developing brain and impair cognitive and behavioral function through multiple mechanisms. The EPA reviewed this evidence and proposed a ban on chlorpyrifos in 2015, citing potential risks posed to women, children, and agricultural communities and workers [7]. The Trump administration reversed the ban earlier this year under "false scientific pretenses," Trasande argues. He calls on scientists to decry such attacks on human health and scientific integrity.

In the absence of a ban on chemicals known to cause harm, one option includes limiting their use around the most vulnerable populations. In California, state officials proposed limiting applications of agricultural pesticides within a quarter of a kilometer of schools and childcare centers after health officials reported that high levels of the chemicals were used near schools. The proposed buffer zone is a step in the right direction, argue Robert Gunier and his colleagues [8]. But a policy designed to safeguard vulnerable populations must account for additive effects of chemical mixtures, the different properties of the wide range of pesticides used in agriculture, and the lack of data to show what distance is truly protective. "The ideal solution to protecting children and pregnant women is an overall reduction in the use of agricultural pesticides to reduce exposure at home and at work, as well as at school," the authors argue.

Chemicals from agriculture, industry, and other commercial uses routinely enter drinking water supplies. One class of chemicals detected in drinking water, called perfluoroalkyl acids

(PFAAs), has come under increased scrutiny because of rapidly emerging evidence that these persistent chemicals accumulate in tissues and cause numerous adverse health effects, even at low levels. Recent research indicates that blood levels of these compounds increase on average by more than 100 times their concentration in drinking water, note Gloria Post and her colleagues [9]. Drinking water guidelines must account for the fact that infants receive much higher exposures than adults from the same drinking water source, and retain these compounds in their bodies years after exposure ends, the authors argue.

As the contributors to this special collection make clear, existing US regulations have not kept pace with scientific advances showing that widely used chemicals cause serious health problems at levels previously assumed to be safe. The most vulnerable population, our children, face the highest risks. More research is needed to better understand the risks posed by these chemicals, identify susceptible groups, and develop safe alternatives. But as the contributors also make clear, science is not always enough. Closing the gap between evidence and policy will require that engaged citizens, both scientists and nonscientists, work to ensure our government officials pass health-protective policies based on the best available scientific evidence.

References

1. Challenges in Environmental Health: Closing the Gap between Evidence and Regulations. http://collections.plos.org/challenges-in-environmental-health
2. Maffini MV, Neltner TG, Vogel S (2017). We are what we eat: Regulatory gaps in the United States that put our health at risk. PLoS Biol 15(12): e2003578. https://doi.org/10.1371/journal.pbio.2003578
3. Krimsky S (2017). The unsteady state and inertia of chemical regulation under the U.S. Toxic Substances Control Act. PLoS Biol 15(12): e2002404. https://doi.org/10.1371/journal.pbio.2002404
4. Lanphear BP (2017). Low-Level Toxicity of Chemicals: No Acceptable Levels? PLoS Biol 15(12): e2003066. https://doi.org/10.1371/journal.pbio.2003066
5. Braun JM, Gray K (2017). Challenges to Studying the Health Effects of Early Life Environmental Chemical Exposures and Children's Health. PLoS Biol 15(12): e2002800. https://doi.org/10.1371/journal.pbio.2002800
6. Trasande L (2017). When Enough Data Are Not Enough to Enact Policy: the Failure to Ban Chlorpyrifos. PLoS Biol 15(12): e2003671. https://doi.org/10.1371/journal.pbio.2003671
7. Federal Register. Chlorpyrifos; Tolerance Revocations; Proposed Rule. Environmental Protection Agency. https://www.gpo.gov/fdsys/pkg/FR-2015-11-06/pdf/2015-28083.pdf Accessed: 6 Nov 2015.
8. Gunier RB, Bradman A, Harley KG, Eskenazi B (2017). Will buffer zones around schools in agricultural areas be adequate to protect children from the potential adverse effects of pesticide exposure? PLoS Biol 15(12): e2004741. https://doi.org/10.1371/journal.pbio.2004741
9. Post GB, Gleason JA, Cooper KR (2017). Key scientific issues in developing drinking water guidelines for perfluoroalkyl acids: Contaminants of emerging concern. PLoS Biol 15(12): e2002855. https://doi.org/10.1371/journal.pbio.2002855
